# Four-dimensional computed tomography is useful for detection of apical sparing of cardiac amyloidosis

**DOI:** 10.1007/s10554-024-03127-6

**Published:** 2024-05-10

**Authors:** Makiko Kinoshita, Hiroyuki Takaoka, Joji Ota, Jun-Ichiro Ikeda, Yoshitada Noguchi, Yusei Nishikawa, Shuhei Aoki, Kazuki Yoshida, Katsuya Suzuki, Satomi Yahima, Haruka Sasaki, Noriko Suzuki-Eguchi, Yoshio Kobayashi

**Affiliations:** 1 Department of Cardiovascular Medicine, Chiba University Graduate School of Medicine, Chiba, Japan; 2 Department of Radiology, Chiba University Hospital, Chiba, Japan; 3 Department of Diagnostic Pathology, Chiba University Graduate School of Medicine, Chiba, Japan

**Keywords:** Computed tomography, Amyloid, Myocardial contraction

## Abstract

A 73-year-old male was admitted because of recurrent syncope. He was diagnosed with transient bradycardia caused by a 2:1 atrioventricular block, and he underwent cardiac computed tomography (CT) using 320 detector-row CT to screen for coronary artery disease. Significant coronary artery stenosis was not detected, but diffuse late iodinate enhancement was found on the epi-myocardium and endo-myocardium of the interventricular septum, and endo-myocardium of the anterior and lateral left ventricular (LV) myocardium (LVM) on CT. The ejection fraction and global longitudinal strain (LS) of LVM were 53.97% and − 9.87% on CT. Apical sparing was present, meaning the LS of LV apical segments were preserved compared with basal segments on CT. Pathological findings of LVM demonstrated loss of myocardial cells and extra-cellular amyloid deposition on the direct fast scarlet staining. He was finally diagnosed with transthyretin amyloidosis.

A 73-year-old male was admitted because of recurrent syncope. He was diagnosed with transient bradycardia caused by a 2:1 atrioventricular block, and he underwent cardiac computed tomography (CT) using 320 detector-row CT (Aquilion ONE/ViSION Edition, Canon Medical Systems, Japan) to screen for coronary artery disease. We performed a non-contrast scan using a prospective electrocardiography (ECG) gating technique and an early-phase scan using a retrospective ECG gating technique for the precise evaluation of coronary arteries [1]. A late-phase scan using a prospective ECG gating technique was performed six minutes after the injection of contrast media. Contrast material was injected using a triphasic protocol as described previously. During the first contrast phase, we injected 70 mL of undiluted iodinated contrast agent (370 mg/mL) at four mL/s, followed by 50 mL of a 50%/50% saline-to-contrast material mixture at four mL/s and 16 mL of pure saline at two mL/s, intravenously [1]. Significant coronary artery stenosis was not detected (Figure A), but diffuse late iodinate enhancement was found on the epi-myocardium and endo-myocardium of the interventricular septum (Figure B - D), and endo-myocardium of the anterior and lateral left ventricular (LV) myocardium (LVM) on CT. The ejection fraction and global longitudinal strain (LS) of LVM were calculated using a new software (Medis Suite CT QStrain 4.2, Medis Medical Imaging, The Netherlands and they were 53.97% and − 9.87% (Figures E - G) (The polar map of LS was presented according to the 17 LVM segments model of the American Heart Association [AHA]). Apical sparing was present, meaning preserved LS of LV apical segments compared with basal segments on CT. LS of LVM was also evaluated using transthoracic echocardiography (Figure H) (The LVM segments of the polar maps for LS were presented with slightly rotated positioning from the 17 LVM segments model of the AHA). Pathological findings of LVM demonstrated loss of myocardial cells and extra-cellular amyloid deposition on the direct fast scarlet staining (Figure 1). He was finally diagnosed with transthyretin amyloidosis.

**Fig. 1:**
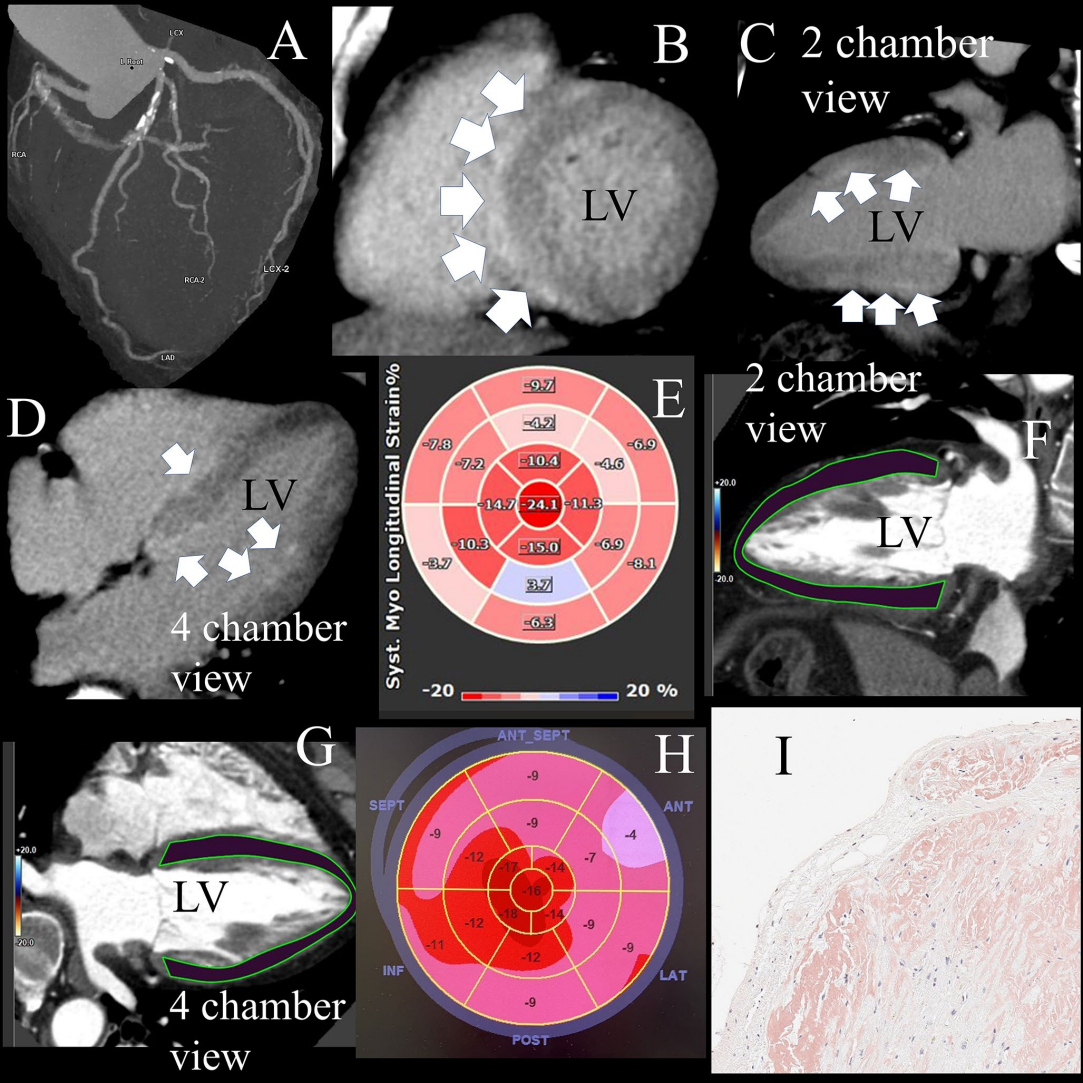
(**A**) Significant coronary artery stenosis was not detected on computed tomography (CT). (**B**–**D**) Diffuse late iodinate enhancement was found on the epi-myocardium and endo-myocardium of the interventricular septum, and endo-myocardium of the anterior and lateral left ventricular (LV) myocardium (LVM) on CT. (**E**–**G**) The ejection fraction and global longitudinal strain (LS) of LVM were calculated using new software (Medis Suite CT QStrain 4.2, Medis Medical Imaging, The Netherlands), and they were 53.97% and − 9.87%. Apical sparing was present, meaning the LS of LV apical segments was preserved compared with basal segments on CT (The polar map of LS was presented according to the 17 LVM segments model of the American Heart Association [AHA]). (**H**) LS of LVM was also evaluated using transthoracic echocardiography (The LVM segments of the polar maps for LS were presented with slightly rotated positioning from the 17 LVM segments model of the AHA). (**I**) Pathological findings of LVM demonstrated loss of myocardial cells and extra-cellular amyloid deposition on the direct fast scarlet staining

Scanning images in a whole cardiac phase differs from the usual protocol of cardiac CT, but scanning images in an entire cardiac phase becomes necessary in cases of arrhythmia. Myocardial strain analysis has been available on CT, and a good correlation was reported between the strain data on CT and echocardiography [2]. Apical sparing is a typical abnormality in patients with cardiac amyloidosis [3]. Furthermore, recent technological innovations in CT equipment have made it possible to significantly reduce radiation doses during cardiac imaging, even with complete cardiac cycle acquisition [4].
